# New therapy with ASC-J9^®^ to suppress the prostatitis *via* altering the cytokine CCL2 signals

**DOI:** 10.18632/oncotarget.11484

**Published:** 2016-08-22

**Authors:** Shin-Jen Lin, Fu-Ju Chou, Chang-Yi Lin, Hong-Chiang Chang, Shuyuan Yeh, Chawnshang Chang

**Affiliations:** ^1^ George Whipple Laboratory for Cancer Research, Departments of Pathology, Urology, and Radiation Oncology, and Wilmot Cancer Center, University of Rochester Medical Center, Rochester, NY 14642, USA; ^2^ Sex Hormone Research Center, China Medical University/Hospital, Taichung 404, Taiwan

**Keywords:** prostate, ASC-J9^®^, CCL2

## Abstract

Prostatitis is a common disease contributing to 8% of all urologist visits. Yet the etiology and effective treatment remain to be further elucidated. Using a non-obese diabetes mouse model that can be induced by autoimmune response for the spontaneous development of prostatitis, we found that injection of the ASC-J9^®^ at 75 mg/Kg body weight/48 hours led to significantly suppressed prostatitis that was accompanied with reduction of lymphocyte infiltration with reduced CD4^+^ T cells in prostate. *In vitro* studies with a co-culture system also confirmed that ASC-J9^®^ treatment could suppress the CD4^+^ T cell migration to prostate stromal cells. Mechanisms dissection indicated that ASC-J9^®^ can suppress CD4^+^ T cell migration *via* decreasing the cytokine CCL2 *in vitro* and *in vivo*, and restoring CCL2 could interrupt the ASC-J9^®^ suppressed CD4^+^ T cell migration. Together, results from *in vivo* and *in vitro* studies suggest that ASC-J9^®^ can suppress prostatitis by altering the autoimmune response induced by CD4^+^ T cell recruitment, and using ASC-J9^®^ may help us to develop a potential new therapy to battle the prostatitis with little side effects.

## INTRODUCTION

Prostatitis is characterized with inflammations in the prostate gland that are classified into acute, chronic, asymptomatic inflammatory prostatitis and chronic pelvic pain syndrome. In the United States, prostatitis is diagnosed in 8% of all urologist visits and 1% of all primary care physician visits [1]. There are four categories of prostatitis [2]: category I includes acute bacterial prostatitis; category II includes chronic bacterial prostatitis, category III includes chronic prostatitis/chronic pelvic pain syndrome (CP/CPPS); and category IV includes asymptomatic inflammatory prostatitis.

CP/CPPS accounts for 90%–95% of prostatitis diagnoses [3]. However, the etiology of this type of prostatitis is still poorly understood. It may result from an interplay between psychological factors and dysfunction in the immune, neurological and endocrine systems [4]. An autoimmune basis for CP/CPPS is a prominent theory for the etiology/pathogenesis of CP/CPPS [5]. Animal models, named as experimental autoimmune prostatitis [6], with the characteristic of CD4-positive T cell infiltrates, have been used as standard *in vivo* models to study the progress of prostatitis.

Markers for cytotoxic T cells are found in the expressed prostatic secretion of men with CP/CPPS, a cell type consistent with autoimmune inflammation [7]. Batstone et al. reported an autoimmune response by looking for T cell proliferation in response to proteins of the seminal plasma (SP) and found SP could increase the T cell proliferation [8]. Ponniah et al. also found that some men with symptoms of chronic prostatitis have evidence of a proliferative CD4-T cell response to prostate specific antigen (PSA) [9].

In the experimental autoimmune prostatitis (EAP) rat model, Morón et al. reported castration could ameliorate the prostatitis symptoms [10], suggesting androgen signaling might play a negative role in developing prostatitis. In the human, the CP/CPPS may be induced by the T cells-induced autoimmune response [8, 9] and the T cells may be linked to alter PSA for the increase cell proliferation.

Here we used the NOD mouse model with spontaneous autoimmune prostatitis [11] to examine the potential roles of androgen receptor (AR), the key regulator of PSA signaling, in the prostatitis that involved the infiltrated T cells. Our results revealed that ASC-J9^®^, a newly developed AR degradation enhancer that could degrade AR in selective cells with few side effects [12–19], could decrease the prostatitis in the NOD mice and suppress the recruitment of T cells to the prostate stromal cells *via* alteration of the cytokine CCL2 signals.

## RESULTS

### ASC-J9^®^ suppresses prostatitis in the spontaneous autoimmune prostatitis mouse

**Figure 1:**
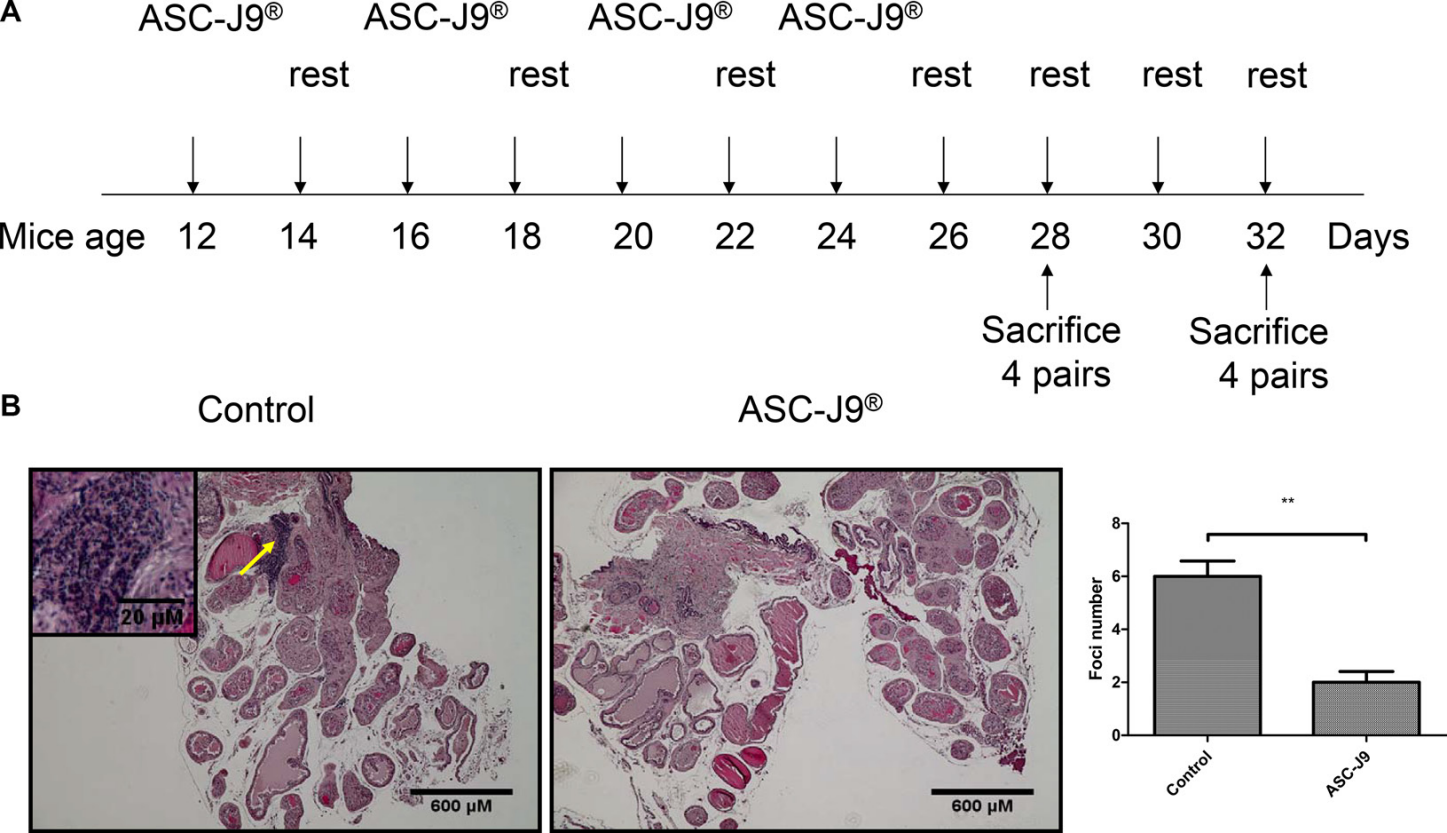
ASC-J9^®^ reduces prostatitis in the NOD mouse (**A**) The scheme shows the time frame of mouse treatment. (**B**) H&E staining of representative prostate sections from ASC-J9^®^ or vehicle control treated NOD mice. A progressively increasing lymphomonuclear cell infiltrate is apparent (arrow). Mean numbers ± SEM of intraprostatic infiltrates/cross section are reported. The statistic analyses were performed using Student *t* test. Each bar represents the mean ± sem. **P < 0.01.

8 pairs of 12-weeks-old NOD mouse that spontaneously develop prostatitis [11] were i.p. injected with 75 mg/kg body weight ASC-J9^®^ or vehicle every 48 hrs for 2 weeks. After 2 weeks of injection, the mice rested for 2 weeks before the next round of injection. The injection/resting cycles were continued until 4 pairs were sacrificed at 28 weeks and the other 4 pairs sacrificed at 32 week of age (see diagram in Figure 1A). Prostate tissues mice were then sacrificed and prostate tissues were collected for H&E staining (Figure 1B, top panel). Autoimmune prostatitis is characterized by lymphoma nuclear cell infiltrates in the prostate gland (indicated by the arrow in Figure 1B). Importantly, ASC-J9^®^ treated mice show reduced infiltrating cells compared to vehicle treated mice. To quantify the extent of prostatitis, intra-prostatic infiltrates foci/cross section were counted and results revealed that ASC-J9^®^ treated mice have reduced foci compared to vehicle treated control mice (Figure 1B, bottom panel), suggesting ASC-J9^®^ could reduce prostatitis in this spontaneous autoimmune prostatitis mouse model.

### ASC-J9^®^ reduces CD4^+^ T cell infiltrates in the NOD mouse

**Figure 2:**
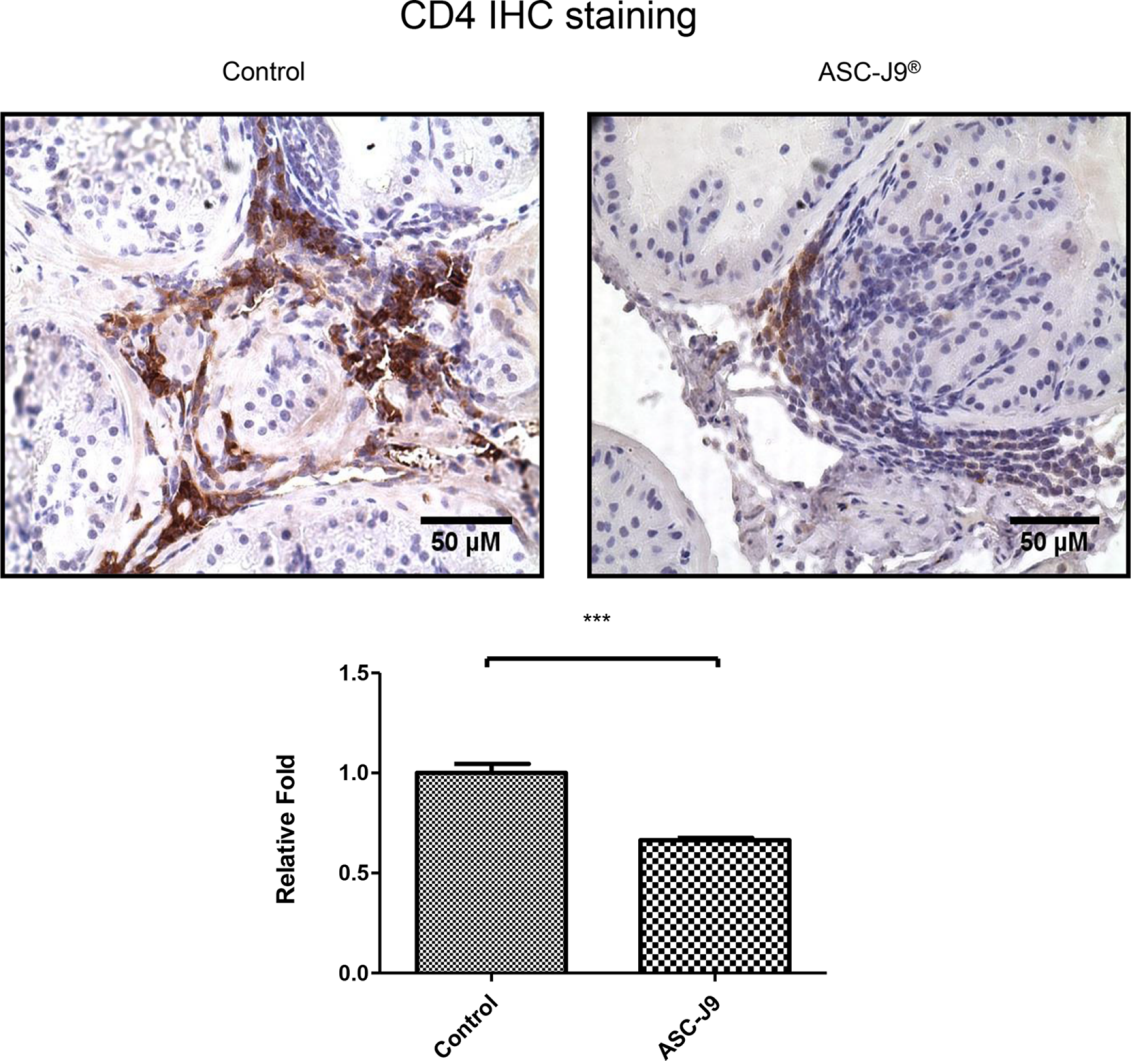
ASC-J9^®^ reduces CD4^+^ T cell infiltrates in the NOD mouse Immunohistochemical staining for CD4 in cross sections of prostates from a pair of representative ASC-J9^®^ or vehicle control treated NOD mice (upper panel). The density of CD4 staining was calculated by ImageJ software by averaging six randomly selected fields (lower panel). The statistic analyses were performed using Student *t* test. Each bar represents the mean ± sem. ***P < 0.001.

To further confirm above *in vivo* mice results, we then assayed the CD4^+^ T cell infiltration to prostate in these NOD mice, as Penna et al. reported the majority of the infiltrating prostatitis cells are CD4^+^ T cells [11]. As shown in Figure 2, ASC-J9^®^ treated NOD mice have less infiltrating CD4^+^ T cells compared to the vehicle treated control mice.

Together, results from Figures 1–2 suggest that ASC-J9^®^ can suppress prostatitis with reduced infiltrated CD4^+^ T cells in the NOD mouse models.

### ASC-J9^®^ reduces CD4^+^ T cell migration to prostate cells in *in vitro* co-culture system

**Figure 3:**
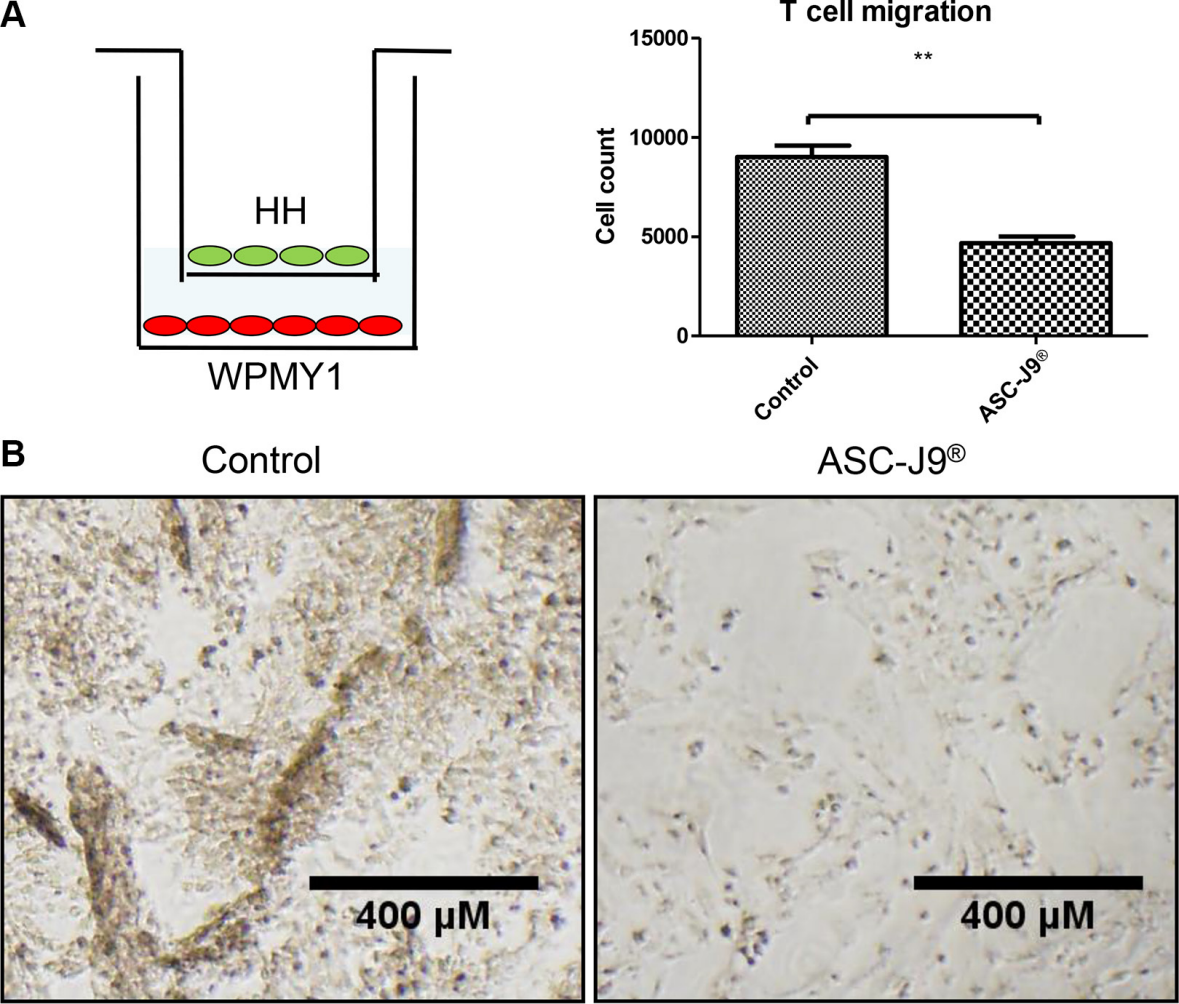
ASC-J9^®^ reduces CD4^+^ T cell migration to the prostate stroma cell (**A**) CD4^+^ T cell HH (top chamber) were co-cultured with prostate stroma cell WPMY-1 (bottom chamber) for the migration assay. The scheme shows the detailed experiment setting of CD4^+^ T cell migration assay (left panel). 2.5 μM of ASC-J9^®^ reduces CD4^+^ T cell migration compared to vehicle control using Boyden chamber transwell migration assay (right panel). (**B**) The pictures show the morphology of WPMY-1 after CD4^+^ T cell recruitment. Left panel shows WPMY-1 started to detach from the culture dish when encountering more migrated CD4^+^ T cells. Right panel shows normal attached WPMY-1 with less CD4^+^ T cells recruited.

We then confirmed the above *in vivo* mice results with *in vitro* co-culture system using migration assay (Figure 3A, left panel). Both HH CD4^+^ T cells and WPMY-1 prostate stromal cells were treated with ASC-J9^®^ 24 hours prior to the migration assay. The results revealed that 2.5 μM of ASC-J9^®^ reduced HH CD4^+^ T cells migration (Figure 3A, right panel). After HH CD4^+^ T cells migrated to the WPMY-1 cells, treatment with ASC-J9^®^ showed little impact on the WPMY-1 cells, whereas in the control group WPMY-1 cells began to show detachment from the plate (Figure 3B). We also observed ASC-J9^®^ can reduce WPMY-1 growth by comparing Figure 3B pictures.

Together, results from above studies suggest that ASC-J9^®^ can suppress infiltrated CD4^+^ T cells migrating to prostate in both *in vitro* co-culture system (Figure 3) and *in vivo* NOD mouse models (Figure 2).

### Mechanism dissection how ASC-J9^®^ reduces CD4^+^ T cell migration to the prostate stromal cells

**Figure 4:**
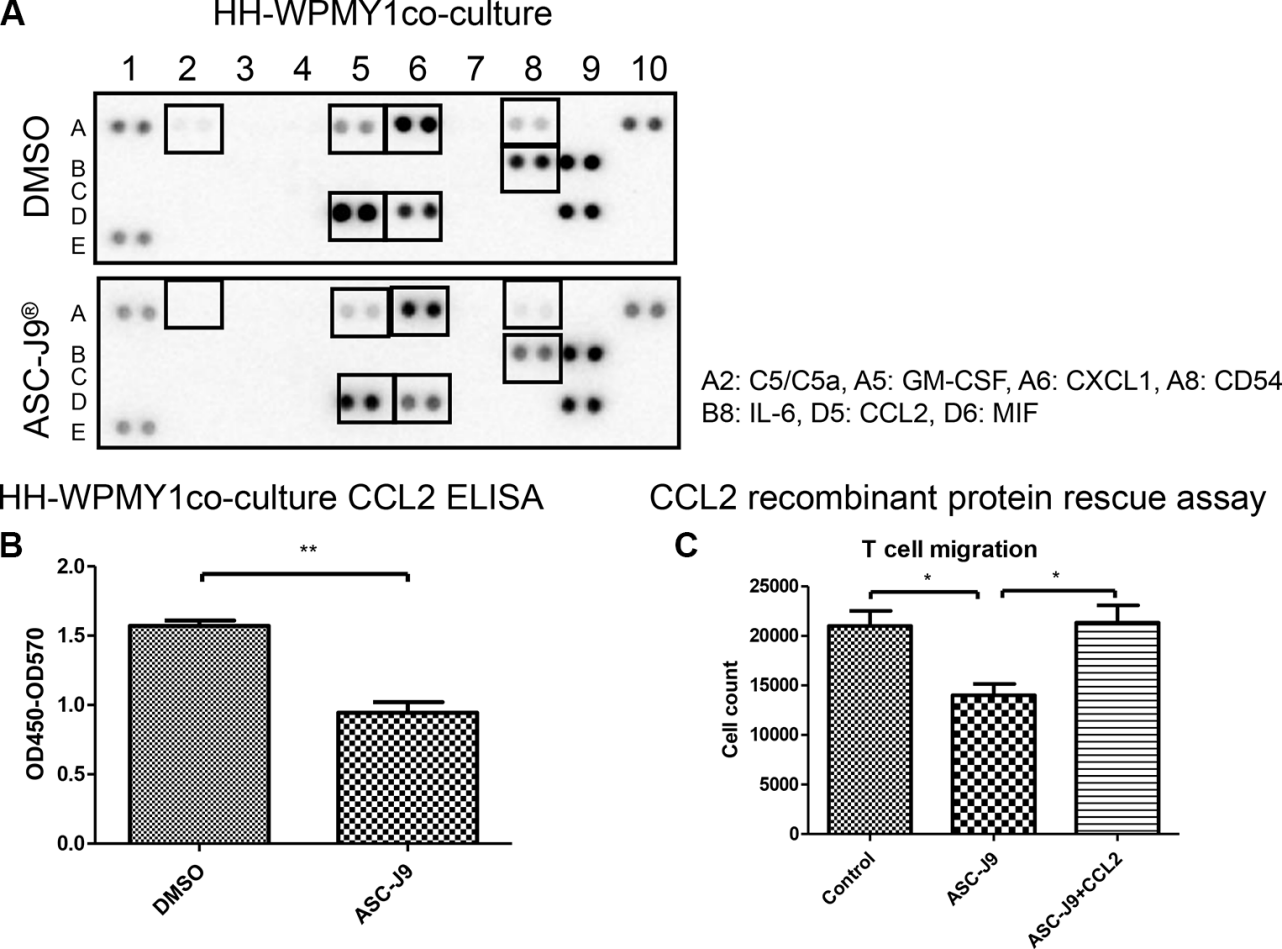
ASC-J9^®^ reduces CD4^+^ T cell migration to the prostate stromal cell through decrease of cytokine CCL2 (**A**) Conditioned media (CM) were collected from WPMY-1 + HH with or without 2.5 μM ASC-J9^®^ treatment for 24 h. Relative amounts of cytokine levels were determined using Human Cytokine Array kit. The cytokines affected by ASC-J9^®^ were denoted by boxes and listed at the right. (**B**) CM collected from co-cultures were also used for detection of CCL2 by human CCL2 ELISA kits. (**C**) The reduction of CCL2 was restored by adding the recombinant CCL2 to the co-culture system. CD4^+^ T cell HH (top well) were co-cultured with prostate stroma cell WPMY-1 (bottom well) for the migration assay. The statistic analyses were performed using Student *t* test. Each bar represents the mean ± sem. *P < 0.05; **P < 0.01.

To further dissect the potential mechanism, we then collected the CM from the co-culture system and arrayed the potential different cytokines/chemokines profile. The results revealed that CCL2 together with other 6 cytokines/chemokines were decreased upon ASC-J9^®^ treatment (Figure 4A), suggesting CD4^+^ T cells migration may be suppressed by ASC-J9^®^ through the reduction of those cytokines/chemokines. Among those altered cytokines, we focused on the CCL2, a key cytokine that can regulate prostate cancer progression yet its expression was suppressed by ASC-J9^®^ [15, 20]. Importantly, early studies also indicated that CCL2 is an essential mediator for the development of prostatitis [21].

**Figure 5:**
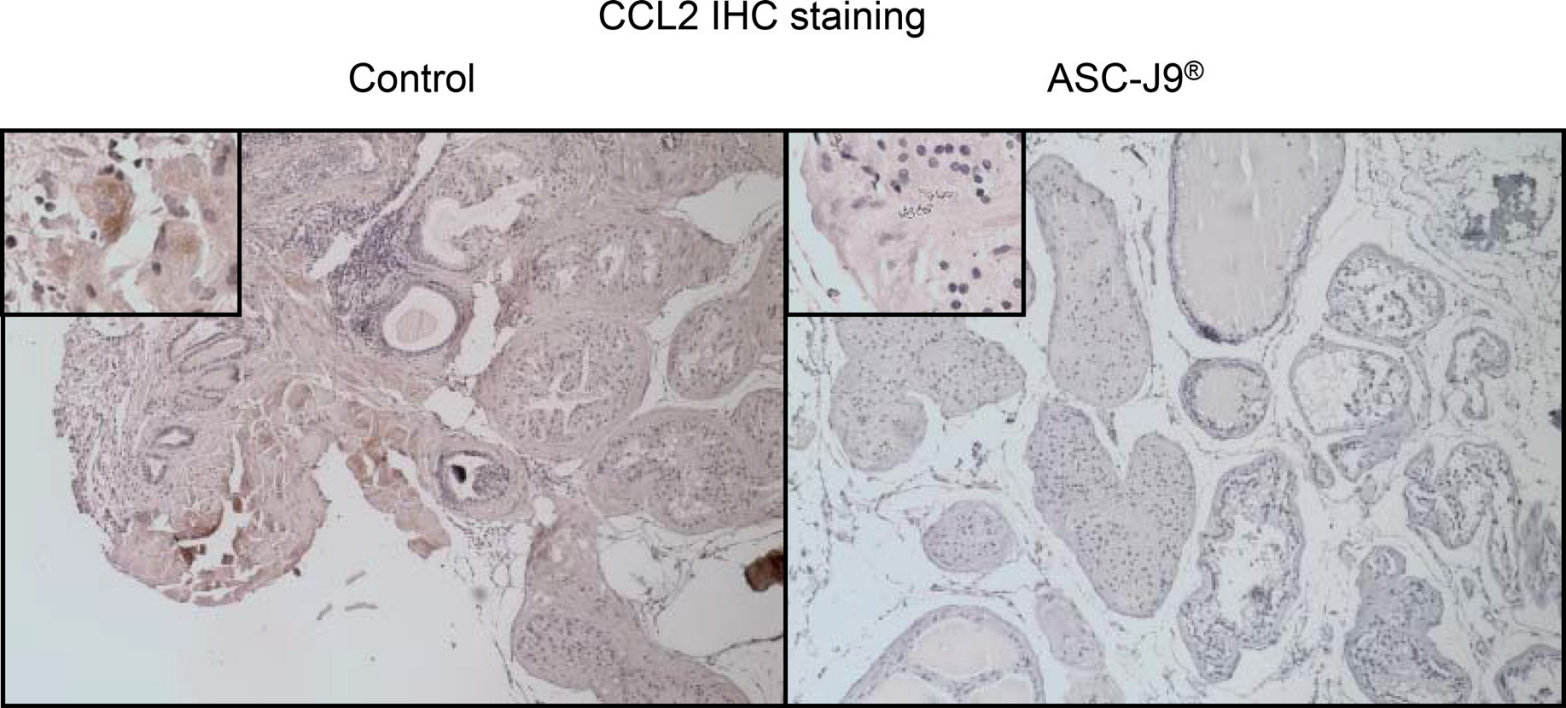
Immunohistochemical staining for CCL2 Immunohistochemical staining for CCL2 in cross sections of prostates from a pair of representative ASC-J9^®^ or vehicle control treated NOD mice. The upper left boxes are 10× magnifications of a representative area of the images.

We first applied the ELISA to confirm that CCL2 is significantly reduced in the HH CD4^+^ T cell and WPMY-1 co-culture system (Figure 4B). We the applied an interruption approach *via* adding the recombinant CCL2 protein to the co-culture system, and found ASC-J9^®^ no longer had the ability to reduce HH CD4^+^ T cells migration, suggesting CCL2 may play a key role in HH CD4^+^ T cells migration to prostate cells (Figure 4C). Importantly, results from *in vivo* mouse model also confirmed the *in vitro* cell co-culture system showing CCL2 was suppressed in the ASC-J9^®^ treated NOD mice compared to the non-treated mice (Figure 5).

Together, both *in vivo* mouse assay (Figures 1–2) and *in vitro* cell migration assay (Figures 3–4) conclude that ASC-J9^®^ can reduce the prostatitis *via* reduction of the CD4^+^ T cell migration to the prostate that involved the modulation of the CCL2 expression (Figures 4–5).

## DISCUSSION

In this study, we demonstrated that infiltrated CD4^+^ T cells play important roles in prostatitis by using NOD mice which can be rescued by ASC-J9^®^, the newly developed AR degradation enhancer. We also demonstrated ASC-J9^®^ can decrease the recruitment of T cells to the prostate stromal cells *via* altering CCL2 cytokine signaling.

ASC-J9^®^ is known to selectively degrade AR in selective cells with very few side effects [12–19]. Importantly, ASC-J9^®^ also functions through a AR-independent pathway to modulate CCL2 in prostate cancer [15]. In this study, HH and WPMY-1 cells used in the *in vitro* co-culture system expressed very little AR. Therefore, we believe ASC-J9^®^ effect may function through a AR-independent pathway to modulate CCL2 in prostatitis. However, AR knockout NOD mice might be better materials to clarify AR role in prostatitis.

Cytotoxic and Helper T cells are reported to be involved in prostatitis [5, 7–9]. Previous studies indicated that tumor-derived CCL2 can enhance progression and malignancy of breast cancer [22, 23]. In contrast, other studies reported that introduction of the CCL2 gene into tumor cells correlated with decreased tumorigenicity and facilitated immune mediated tumor rejection [24–26]. These studies all suggested that CCL2 plays important roles in immune cells infiltration and inflammatory responses. In this study, we found CCL2 has a key role in CD4^+^ T cells recruitment and causes inflammatory reactions in prostate. ASC-J9^®^ might be a potential anti-inflammation drug *via* altering CCL2 expression to reduced inflammatory immune cell infiltration.

Together, this study suggests a small molecule, ASC-J9^®^, can suppress prostatitis *via* the AR-independent pathway. Future successful clinical trials with ASC-J9^®^ to suppress prostatitis may provide us a new and better therapy to battle prostatitis.

## MATERIALS AND METHODS

### Animals and reagents

NOD mice were from The Jackson Laboratory. Mice were kept under specific pathogen-free conditions. All animal studies have been approved by the institutional review board of the University of Rochester Medical Center Department of Laboratory and Animal Medicine. CCL2 recombinant protein was purchased from R&D Systems.

### ASC-J9^®^ injection

NOD mouse that spontaneously develop prostatitis were i.p. injected with 75 mg/kg body weight ASC-J9^®^ or vehicle every 48 hrs for 2 weeks. After 2 weeks of injection, the mice rested for 2 weeks before next round of injection. The injection/resting cycles were continued until 4 pairs were sacrificed at 28 weeks and the other 4 pairs sacrificed at 32 week of age.

### IHC staining

Mouse prostate tissues were collected and fixed by 10% formalin followed by paraffin embedding. Samples were sliced to 5 μm thickness. We used the primary antibodies of anti-CD4 (BD Biosciences) and anti-CCL2 (Biolegend). The primary antibody was recognized by the biotinylated secondary antibody and visualized by Vectastain ABC peroxidase system and peroxidase substrate DAB kit (Vector Laboratories).

### Cell culture

T-lymphocytic cell line HH (CD4+) and prostate stroma cell line WPMY-1 were acquired from the American Type Culture Collection (ATCC) and maintained in RPMI 1640 medium (GIBCO) supplemented with 10% fetal bovine serum and 1% Antibiotic-Antimycotic solution (Invitrogen).

### T cell recruitment assay

HH and WPMY-1 were treated with vehicle or 2.5 μM of ASC-J9^®^ for 1 day. Recruitment assay was performed using 24-well transwell inserts (8 μm pores) according to the manufacturer's instructions (Corning, #3422). ASC-J9^®^ treated HH cells were seeded on the upper chambers of transwell plates and WPMY-1 cells were seeded on the lower chambers of transwell plates containing 20% FBS. Migrated HH cells were counted after 24 h incubation. Each sample was assayed in triplicate.

### Human cytokine antibody array and ELISA

Condition media CM were collected from WPMY-1 + HH cells with or without 2.5 μM ASC-J9^®^ treatment for 24 h. Relative amounts of cytokine levels were determined using Human Cytokine Array kit (Panel A, ARY005, R&D Systems) according to the manufacturer's instructions. CM collected from co-cultures were also used for detection of CCL2 by human CCL2 ELISA kits (R&D Systems) according to the manufacturer's instructions.

### Statistics

The data values were presented as the mean ± sem. *P* values were calculated by unpaired Student's *t* test or Fisher's exact test. *P* < 0.05 was considered statistically significant.
